# Trends in public sector radiological usage in the Western Cape Province, South Africa: 2009–2019

**DOI:** 10.4102/sajr.v25i1.2251

**Published:** 2021-11-30

**Authors:** Monica van Wijk, Michelle M. Barnard, Amanda Fernandez, Keith Cloete, Matodzi Mukosi, Richard D. Pitcher

**Affiliations:** 1 Division of Radiodiagnosis, Department of Medical Imaging and Clinical Oncology, Faculty of Medicine and Health Sciences, Stellenbosch University and Tygerberg Hospital, Cape Town, South Africa; 2 Sub-Directorate Medical Imaging Services, Directorate: Health Technology, Western Cape Department of Health, Cape Town, South Africa; 3 Tygerberg Hospital, Department of Health, Western Cape Government, Cape Town, South Africa

**Keywords:** radiology, trends, utilisation, middle-income country, healthcare, public sector

## Abstract

**Background:**

Although global use of medical imaging has increased significantly, little is known about utilisation trends in low- and middle-income countries (LMICs).

**Objectives:**

To evaluate changes over a decade in public sector diagnostic imaging utilisation at provincial level in a middle-income country.

**Method:**

A retrospective analysis of medical imaging utilisation in the Western Cape Province of South Africa in 2009 and 2019. Use of conventional radiography, ultrasonography (US), fluoroscopy, CT, MRI, digital subtraction angiography (DSA) and whole-body digital radiography was assessed by total studies and studies/10^3^ people, for the whole province, the rural and metropolitan areas. Mammography utilisation was calculated for every 10^3^ females aged 40–70 years.

**Results:**

The provincial population and total imaging investigations increased by 25% and 32%, respectively, whilst studies/10^3^ people increased by 5.5% (256 vs 270/10^3^), with marked variation by modality. Provincial US, CT and MRI utilisation/10^3^ people increased by 111% (20 vs 43/10^3^), 78% (10 vs 18/10^3^) and 32% (1.9 vs 2.5/10^3^) respectively, whilst use of fluoroscopy (3.6 vs 3.7/10^3^) and mammography (14.2 vs 15.9/10^3^ women aged 40–70 years) was steady and plain radiography decreased by 20% (216 vs 196/10^3^). For CT, mammography and fluoroscopy, percentage utilisation increases/10^3^ people were higher in the rural than metropolitan areas.

**Conclusion:**

Population growth is the main driver of overall imaging utilisation in our setting. The relatively constant imaging workload per 1000 people, albeit with increasing ultrasound, CT and MR utilisation, and decreasing use of plain radiography, reflects improved provincial imaging infrastructure, and appropriate use of available resources.

## Introduction

There have been remarkable advances in diagnostic imaging since the discovery of X-rays just over 120 years ago, with the evolution of fluoroscopy, angiography, mammography and CT. Additionally, in the last 60 years, modalities that do not utilise ionising radiation, namely ultrasound (US) and magnetic resonance (MR), have emerged, whilst progress in information technology in the last three decades has yielded picture archiving and communication systems (PACS), radiology information systems (RIS), fully digital, paperless radiology departments and teleradiology. These developments have entrenched diagnostic imaging as a pivotal clinical service.^1,2,3,4^

Between 1988 and 2016, annual global imaging investigations increased by 161% from 1.38 to 3.6 billion studies, representing an average annual increase of approximately 6%.^5,6^ However, the growing global demand for imaging is a challenge, since radiological services are capital and labour intensive, demand high levels of technical expertise and may involve the hazards of ionising radiation.^4,5,6,7^ Despite medical imaging currently accounting for approximately 10% of the total per capita healthcare expenditure in well-resourced environments, radiological needs are perceived to remain unmet.^8,9,10^ Increased utilisation is predominantly in the more sophisticated modalities.^3,4,11^ In the United Kingdom, from 2012 to 2020, the average annual CT, MR, US and X-ray utilisation increased by 10%, 9%, 5% and 1%, respectively.^12^ Between 2003 and 2019, MR utilisation in Canada and the United States showed an average annual increase of 10% and 5%, respectively, whilst the corresponding average annual CT usage increased by 7% and 5%, respectively.^13^

Advances in imaging technology have the potential to compound inequity in global access to services. Well-resourced environments, with an aging population and a high prevalence of non-communicable diseases tend to have a ready supply of trained imaging personnel and state-of-the-art equipment, but are confronted by over-utilisation, unsustainable imaging consumption and an escalating population exposure to ionising radiation.^5,14,15,16^ Conversely, many low- and middle-income countries (LMICs) have a disease burden largely related to poverty and lack of access to basic medical imaging, particularly in the rural areas.^17,18,19^ Of note, Organisation for Economic Cooperation and Development (OECD) countries average 14 MRI and 25 CT units per million people, compared to an average of 0.1 MRI and 0.6 CT units per million people in Southern and East African countries.^20,21,22^ Increased imaging resources are associated with increased utilisation of radiological services. In the last decade, an estimated 1245 imaging investigations per 1000 people were performed in the United States, of which 949 made use of ionising radiation, including 149 CT scans; European Commission countries averaged 505 ionising radiation investigations per 1000 people, including 79 CT scans; OECD countries averaged 144 CT scans per 1000 people.^4,13,23^

By contrast, an analysis of imaging utilisation in the public healthcare sector of South Africa’s (SA) Western Cape Province (WCP) in 2017 documented 262 radiological examinations per 1000 people, 218 utilising ionising radiation, including 16 CT scans.^24^

However, little is known about temporal trends in LMIC public-sector imaging utilisation. It is acknowledged that analyses of registered diagnostic imaging equipment in resource-constrained environments provide useful insights into healthcare access and equity.^25,26^ Trends in imaging utilisation allow appreciation of advances in LMIC healthcare infrastructure, assist in defining population-based norms, guide healthcare planning and policy and potentially serve as a yardstick for sustainable imaging practice. Such knowledge is also pivotal for the assessment of population radiation exposure, a focus of ongoing analysis by the United Nations Educational, Scientific and Cultural Organisation.^5^

South Africa is one of just five upper middle-income countries in sub-Saharan Africa. Public sector healthcare is delivered by the District Health System (DHS) and administered at provincial level.^27^ The WCP is the most southern of SA’s nine provinces and comprises six managerial districts. The Cape Town Metropolitan District, with more than 60% of the provincial population, but just 2% of the land area, is surrounded by five rural districts.^27,28,29^ Accordingly, the metropolitan population density exceeds the rural population density by a factor of almost 90 (1682 vs 19 people/km^2^; 89:1).^24^

Western Cape Province health services are based on mirrored, tiered referral pathways for the metropolitan and rural areas.^30^ Initial imaging access is typically at community centres with subsequent referral to district, regional and central hospitals with progressive access to more specialised imaging modalities. The central hospitals are university-affiliated tertiary-level teaching institutions.

The WCP public sector healthcare infrastructure was considered ideal for the analysis of diagnostic imaging utilisation patterns. There is a digital imaging platform across the WCP, with PACS-integration of services at the various levels of care. This eliminates unnecessary duplication of services by facilitating access to imaging by the various healthcare facilities across the platform. The Medical Imaging Services Sub-Directorate (MISSD) within the Directorate of Health Technology (DOHT) in the WCP Department of Health (DoH) is tasked with collation of all data pertaining to the utilisation of provincial diagnostic imaging services.

The aim of this study was to analyse changes over a decade in the utilisation of public sector diagnostic imaging services at the provincial level in a middle-income country.

## Materials and methods

This was a retrospective audit of the utilisation of public sector radiological services in the WCP of SA in 2009 and 2019.

Imaging utilisation data for 2009 and 2019 were extracted from the database of the MISSD and stratified by imaging modality (plain radiographs, US, fluoroscopy, mammography, angiography, CT, MR) and by geographic location (rural/metropolitan). Population statistics for 2009 and 2019 were obtained from the District Development Model documentation of the Department of Cooperative Governance and Traditional Affairs and supplied by Information Handling Services (IHS) Markit (personal communication, J Boshoff). Approximately 75% of the WCP population is dependent on public healthcare.^31^ Imaging studies performed per 1000 people reliant on the public healthcare sector were calculated by modality for 2009 and 2019, for the whole province, and for the metropolitan and rural areas.^29,31^ The ratio of metropolitan: rural studies performed across the modalities was compared for 2009 and 2019. Magnetic resonance, digital angiography and Lodox whole-body scanning were considered shared/central services.

For the analysis, plain radiographs were evaluated in total, as well as by chest X-rays and general X-rays, the latter being all plain radiographs other than chest X-rays. Workload was analysed by an absolute number of investigations and investigations per 1000 people. For breast imaging, analysis was by mammograms per 1000 women aged 40–70 years, the screening age recommended by the Radiological Society of South Africa as well as the Breast Imaging Society of South Africa.^27,32,33^

### Ethical considerations

The study was approved by the Health Research Ethics Committee of the Faculty of Medicine and Health Sciences at Stellenbosch University, and by the Health Research Committee of the WCP, under the auspices of the National Health Research Database (project reference 1703; HREC reference: N17/10/098). It was undertaken with the full support of the Head of Health of the Western Cape Government (WCG) and the Imaging Task Team of the WCG DoH.

## Results

### Provincial analysis

#### Population

Between 2009 and 2019, the WCP population grew by 25% (*n* = 1.36 million people; 5.45 million vs 6.81 million).

#### Overall imaging utilisation

In the same period, the annual provincial imaging workload increased by 32% (*n* = 333 807; 1.05 million vs 138 million), with increments across all modalities.

Ultrasound showed the highest numerical (*n* = 135 570) and percentage (164%) growth, whilst CT, MR and mammography utilisation grew by 123%, 62% and 59%, respectively.

Plain X-rays were the most common investigation throughout the review period (884 981 vs 1 005 545) but showed the lowest percentage growth (14%). In 2009, plain X-rays represented 83% of all imaging investigations, compared to 73% in 2019. By contrast, US increased from 8% to 16% of all provincial imaging investigations.

#### Imaging utilisation per 1000 people

Between 2009 and 2019, the number of imaging investigations/10^3^ people increased from 256 to 270, representing 5% of overall growth, or an average annual increment of just 1.4 studies/10^3^ people. Utilisation trends varied across modalities. (see Tables 1 and 2).

**TABLE 1:** Overall utilisation.

Variable	Metropolitan								Rural								Province							
	2009		2019		Total increase (*n*)	Average annual increase (*n*)	% increase	Average annual % increase	2009		2019		Total increase (*n*)	Average annual increase (*n*)	% increase	Average annual % increase	2009		2019		Total increase (*n*)	Average annual increase (*n*)	% increase	Average annual % increase
	*n*	%	*n*	%																				
					*n*	%							*n*	%	*n*	%							*n*	%
Total population	3 478 914	-	4 392 562	-	913 648	91 364.8	26.3	2.6	1 971 757	-	2 416 387	-	444 630	44 463.0	22.5	2.3	5 450 671	-	6 808 949	-	1 358 278	135 827.8	24.9	2.5
Population reliant on public healthcare	2 619 622	-	3 307 599	-	687 977	68 797.7	26.3	2.6	1 484 733	-	1 819 539	-	334 806	33 480.6	22.5	2.3	4 104 355	-	5 127 139	-	1 022 784	102 278.4	24.9	2.5
Female population 40–70 years	-	-	-	-	-	-	-	-	-	-	-	-	-	-	-	-	732 900	-	1 037 588	-	304 688	30 468.8	41.6	4.2
Females 40–70 reliant on public healthcare	-	-	-	-	-	-	-	-	-	-	-	-	-	-	-	-	551 874	-	781 304	-	229 430	22 943.0	41.6	4.2
Chest X-ray	314 183	44.7	333 624	35.5	19 441	1944.1	6.2	0.6	136 267	41.4	137 658	33.6	1391	139.1	1.0	0.1	450 450	42.9	471 282	34.0	20 832	2083.2	4.6	0.5
General X-ray	274 107	38.9	351 127	37.4	77 020	7702.0	28.1	2.8	160 424	48.7	183 136	44.7	22 712	2271.2	14.2	1.4	434 531	41.3	534 263	38.6	99 732	9973.2	23.0	2.3
Total X-ray	588 290	83.7	684 751	72.9	96 461	9646.1	16.4	1.6	296 691	90.1	320 794	78.3	241 03	2410.3	8.1	0.8	884 981	84.2	1 005 545	72.6	120 564	12 056.4	13.6	1.4
Ultrasound	52 522	7.5	147 389	15.7	94 867	9486.7	180.6	18.1	30 257	9.2	70 961	17.3	40 704	4070.4	134.5	13.5	82 779	7.9	218 350	15.8	135 571	13 557.1	163.8	16.4
Fluoroscopy	13 285	1.9	16 644	1.8	3359	335.9	25.3	2.5	1504	0.5	2396	0.6	892	89.2	59.3	5.9	14 789	1.4	19 040	1.4	4251	425.1	28.7	2.9
Mammography	6971	1.0	10 772	1.1	3801	380.1	54.5	5.5	844	0.3	1644	0.4	800	80.0	94.8	9.5	7815	0.7	12 416	0.9	4601	460.1	58.9	5.9
CT	41 895	6.0	79 255	8.4	37 360	3736.0	89.2	8.9	98	0.0	14 164	3.5	14 066	1406.6	14 353.1	1435.3	41 993	4.0	93 419	6.7	51 426	5142.6	122.5	12.3
MR	-	-	-	-	-	-	-	-	-	-	-	-	-	-	-	-	7808	0.7	12 641	0.9	4833	483.3	61.9	6.2
Digital subtraction angiography	-	-	-	-	-	-	-	-	-	-	-	-	-	-	-	-	10 969	1.0	17 441	1.3	6472	647.2	59.0	5.9
Lodox	-	-	-	-	-	-	-	-	-	-	-	-	-	-	-	-	0.0	0.0	6089	0.4	6089	608.9	x	x
Total studies	702 963	-	938 811	-	235 848	23 584.8	33.6	3.4	329 394	-	409 959	-	80 565	8056.5	24.5	2.5	1 051 134	-	1 384 941	-	333 807	33 380.7	31.8	3.2

**TABLE 2:** Utilisation per 1000 people.

Variable	Metropolitan						Rural						Province					
	2009	2019	Total increase (*n*/1000 people)	Average annual increase (*n*/1000 people)	Total % increase per 1000 people	Average annual % increase per 1000 people	2009	2019	Total increase (*n*/1000 people)	Average annual increase (*n*/1000 people)	Total % increase per 1000 people	Average annual % increase per 1000 people	2009	2019	Total increase (*n*/1000 people)	Average annual increase (*n*/1000 people)	Total % increase per 1000 people)	Average annual % increase per 1000 people)
Chest X-ray	119.9	100.9	−19.0	−1.9	−15.8	−1.6	91.8	75.7	−16.1	−1.6	−17.5	−1.8	109.7	91.9	−17.8	−1.8	−16.2	−1.6
General X-ray	104.6	106.2	1.6	0.2	1.5	0.2	108.0	100.6	−7.4	−0.7	−6.9	−0.7	105.9	104.2	−1.7	−0.2	−1.6	−0.2
Total X-ray	224.6	207.0	−17.6	−1.8	−7.8	−0.8	199.8	176.3	−23.5	−2.4	−11.8	−1.2	215.6	196.1	−19.5	−2.0	−9.0	−0.9
Ultrasound	20.0	44.6	24.6	2.5	123.0	12.3	20.4	39.0	18.6	1.9	91.2	9.1	20.2	42.6	22.4	2.2	110.9	11.1
Fluoroscopy	5.1	5.0	−0.1	0.0	−2.0	−0.2	1.0	1.3	0.3	0.0	30.0	3.0	3.6	3.7	0.1	0.0	2.8	0.3
Mammography†	2.7	3.3	0.6	0.1	22.2	2.2	0.6	0.9	0.3	0.0	50.0	5.0	1.9 (14.2)	2.4 (15.9)	0.5 (1.7)	0.1 (0.2)	26.3 (12.0)	2.6 (1.2)
CT	16.0	24.0	8.0	0.8	50.0	5.0	0.07	7.8	7.7	0.8	11 000.0	1100.0	10.2	18.2	8.0	0.8	78.4	7.8
MR	-	-	-	-	-	-	-	-	-	-	-	-	1.9	2.5	0.6	0.1	31.6	3.2
Digital subtraction angiography	-	-	-	-	-	-	-	-	-	-	-	-	2.7	3.4	0.7	0.1	25.9	2.6
Lodox	-	-	-	-	-	-	-	-	-	-	-	-	0.0	1.2	1.2	0.1	x	x
Total studies per 1000	268.3	283.8	15.5	1.6	5.8	0.6	221.9	225.3	3.4	0.3	1.5	0.2	256.1	270.1	14	1.4	5.5	0.6

CT, computed tomography; MR, magnetic resonance.

† , mammography figures for women 40–70 years of age indicated in brackets.

Ultrasounds/10^3^ people more than doubled (20 vs 43; 111%) whilst CT utilisation increased almost 80% (10 vs 18; 78%) and that of MR by nearly one third (1.9 vs 2.5; 32%).

The use of fluoroscopy (3.6 studies/10^3^ people) and mammography (14.2 vs 15.9 studies/10^3^ women aged 40–70 years) was steady, whilst that of general radiography declined 14%, from 216 to 196 studies/10^3^ people.

### Metropolitan versus rural analysis

#### Population

The City of Cape Town population growth of 26% (*n* = 913 000; 3.48 million vs 4.39 million) was slightly higher than the 23% (*n* = 444 630; 1.97 million vs 2.42 million) recorded in the rural districts. In 2009, 63.8% of the WCP population lived in the City of Cape Town, compared to 64.5% in 2019.

#### Overall imaging utilisation

The rural districts recorded higher percentage increases in the more sophisticated imaging modalities such as CT, fluoroscopy and mammography. This was particularly true for CT, where rural workload increased 144–fold (*n* = 14 066; 98 vs 14 164).

#### Imaging utilisation per 1000 people

Metropolitan use of the more sophisticated imaging modalities was higher than the rural use throughout the review period, although differences were smaller in 2019. For example, CT utilisation per 10^3^ people was 240 times higher in the metropole in 2009, compared to 3-times higher in 2019.

There was substantial and comparable growth in US usage in the metropolitan and rural districts, whilst chest radiography declined in similar measure in both metropolitan and rural districts.

## Discussion

To our knowledge, this is the most detailed analysis of utilisation trends for public sector radiological services in either a low- or a middle-income country. It, therefore, represents a key reference, contributing to crucial discussions on equitable access to healthcare and appropriate and sustainable utilisation of diagnostic imaging in less resourced environments. It can serve as a benchmark and stimulate similar work in this domain. There are five key findings.

*Firstly,* whilst the provincial population expanded 25% and the overall radiological workload increased 32%, the corresponding increment in investigations per 1000 people was just 5%, suggesting that population growth is the main driver of overall imaging utilisation in our setting.

*Secondly*, whilst there was relatively little change in the number of studies per 1000 people, there was marked variation in utilisation by modality, broadly characterised as increased use of US, CT and MR, and decreased recourse to plain radiography.

*Thirdly*, the work provides key health system planning data for population growth, showing that for every 1000 people, healthcare infrastructure is required for approximately 260 imaging investigations per annum, if overall provincial access to radiological services is to be maintained.

*Fourthly*, the study highlights the importance of invoking dual analyses when assessing healthcare utilisation trends. Both the absolute number of patient interactions and the number of patient interactions per 1000 people are key utilisation indicators.

*Fifthly,* in our setting, and across all modalities, utilisation of imaging services per 1000 people remains substantially lower than that documented in well-resourced environments.

The WCP population expansion of 25% in the review period was higher than the South African national average of 19% and was impacted by migration and improved health indicators.^27,32^ Between 2006 and 2021, the WCP had South Africa’s second-highest provincial in-migration (after Gauteng), gaining almost 900 000 inhabitants through interprovincial or international relocation.^27^ Additionally, key health indicators improved in the review period, with 5.8% and 7.5% increased life expectancy for females (67.2 vs 71.1 years) and males (61.1 vs 65.7 years), respectively, 75% reduction in mother-to-child HIV-transmission (11.8% in 2008 vs 3.4% in 2018), 38% reduction in mortality amongst children aged 1–59 months and 29% decrease in neonatal deaths.^27,34^

The finding that radiological workload tends to be diverted from plain radiography, with increasing availability of newer modalities such as US, CT and MRI, is intuitive. For many years, observers have noted that the number of providers of a given medical service is closely related to the rate at which the service is used.^35,36^ However, to date, this trend has not been formally documented or quantified for diagnostic imaging. The demonstration that the number of radiological investigations per 1000 people remains relatively constant, despite the increasing availability of newer modalities is of particular interest. This suggests prudent use of WCP radiological resources. It is in line with Smith-Bindman’s contention that newer imaging tests should replace, not supplement, older, less accurate modalities, in the interest of cost containment.^3^

Trends in WCP imaging utilisation by modality reflect the expansion of provincial radiological infrastructure in the review period. Key developments included the introduction of three new rural and four new metropolitan CT services, the commissioning of a new rural mammography service and a 50% increase in the operating hours of one provincial MR facility. Additional US services were introduced at seven sites between 2015 and 2019 (49 vs 56 centres) and basic medical imaging services were introduced in five healthcare centres. For each new service, appropriate additional radiologist, radiographer and sonographer resources were recruited.^37,38^

**FIGURE 1:**
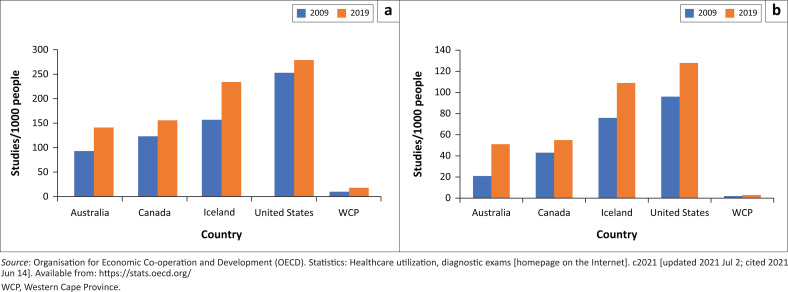
Comparison of Western Cape Province imaging trends with published international trends, 2009–2019. (a) Comparison of computed tomography utilisation, (b) comparison of magnetic resonance imaging utilisation.

This study also provides novel insights into the utilisation of WCP radiological services, compared to high-income countries (Figures 1). Western Cape Province plain radiograph utilisation (216 vs 196 studies/10^3^ people in 2009 and 2019, respectively) was less than one third of the United States (722/10^3^ people) in 2010 and approximately half of European usage (410/10^3^ people) in 2008.^4,23^ Local US workload (20 vs 40 studies/10^3^ people) from 2009 through 2019 was consistently 8–9 fold less than the United States (177 vs 347/10^3^ people) and Canada (188 vs 386/10^3^ people) between 2000 and 2016.^39^ Western Cape Province CT studies in 2009 and 2019 (10 vs 18.2/10^3^; 78% increase) were approximately one sixteenth and one tenth of average figures for the United States (252 vs 278/10^3^; 10% increase), Canada (123 vs 156/10^3^; 27% increase) and Australia (93 vs 141/10^3^; 52% increase) in the same years, respectively.^13^ Similarly, WCP MRI workload during the review period (1.9 vs 2.5 studies/10^3^ people; 32% increase) remained, on an average, 26 times lower than the corresponding annual figures for the United States (96 vs 128/10^3^ people; 33% increase), Canada (43 vs 55/10^3^ people; 28% increase) and Australia (21 vs 51/10^3^ people; 143% increase).^13^ Local mammography utilisation (15 studies/10^3^ women) in the eligible population is approximately 45-times lower than in the United Kingdom.^40^

A major strength of this study is the consistent and meticulous collation of imaging utilisation data by a central provincial body constituted for a specific purpose. The reported data are unique in the South African context, and to our knowledge, across all LMICs. A weakness is the absence of detail regarding the clinical indication for imaging, specifics of imaging protocols, particularly the anatomical region of studies and demographic particulars of the imaged population. Going forward, consideration should be given to collating these additional data, which could enhance the understanding of the complex relationship between burden of disease and imaging utilisation. A further limitation is the absence of detailed corresponding data on provincial equipment and personnel resources through the review period. Data pertaining to the radiological workforce are available from the register of the Health Professions Council of South Africa (HPCSA). In the WCP, between 2011 and 2019, registered sonographers increased by 135% (46 vs 108), registered radiographers increased by 57% (795 vs 1247) and registered radiologists increased by 26% (186 vs 234). However, these numbers include both the public and private sectors and reflect the address at the time of HPCSA registration, rather than on-going employment in the WCP.^37^ Further detailed data would have contributed to a more insightful analysis of the impact of improved imaging infrastructure on utilisation of resources. Of note, since 2014, detailed data on provincial equipment and personnel have been collated and can be correlated with utilisation trends in future analyses. An additional limitation is the potential under-reporting of US studies. Such investigations are increasingly performed by medical practitioners outside the domain of diagnostic imaging and are not reported in this analysis. A final limitation was the inability to accurately stratify the female population aged 40–70 years by geographical region (metropolitan/rural). Accordingly, the analysis of mammography utilisation was for the province as a whole.

It is hoped that this work will serve as a yardstick for analyses of imaging utilisation in resource-limited settings and will stimulate further work in this domain, particularly in other South African provinces.

## Conclusion

From 2009 to 2019, imaging use in the public healthcare sector of the WCP increased in total, with the greatest increase in the advanced modalities, which have correspondingly become more accessible to rural populations. Imaging utilisation rate per 1000 was maintained at approximately 260 studies, indicating that population growth was the main driver of the increase in imaging. This number provides an important yardstick for sustainable imaging practice and overall access needs of an LMIC. Total and population-based frequency of imaging is substantially lower than that of well-resourced settings.
